# Cross-presentation of a TAP-independent signal peptide induces CD8 T immunity to escaped cancers but necessitates anchor replacement

**DOI:** 10.1007/s00262-021-02984-7

**Published:** 2021-06-17

**Authors:** Koen A. Marijt, Lisa Griffioen, Laura Blijleven, Sjoerd. H. van der Burg, Thorbald van Hall

**Affiliations:** Department of Medical Oncology, C7-P, Oncode Institute, Leiden University Medical Center, Albinusdreef 2, 2333 ZA Leiden, The Netherlands

**Keywords:** Immunotherapy, Synthetic long peptide (SLP) vaccine, Immune escape, Transporter associated with antigen processing (TAP), Cross-presentation, Dendritic cells

## Abstract

Cancer cells frequently display defects in their antigen-processing pathway and thereby evade CD8 T cell immunity. We described a novel category of cancer antigens, named TEIPP, that emerge on cancers with functional loss of the peptide pump TAP. TEIPPs are non-mutated neoantigens despite their ‘self’ origin by virtue of their absence on normal tissues. Here, we describe the development of a synthetic long peptide (SLP) vaccine for the most immunogenic TEIPP antigen identified thus far, derived from the TAP-independent LRPAP1 signal sequence. LRPAP1_21–30_-specific CD8 T cells were present in blood of all tested healthy donors as well as patients with non-small cell lung adenocarcinoma. SLPs with natural flanking, however, failed to be cross-presented by monocyte-derived dendritic cells. Since the C-terminus of LRPAP1_21–30_ is an unconventional and weakly binding serine (S), we investigated if replacement of this anchor would result in efficient cross-presentation. Exchange into a valine (V) resulted in higher HLA-A2 binding affinity and enhanced T cell stimulation. Importantly, CD8 T cells isolated using the V-variant were able to bind tetramers with the natural S-variant and respond to TAP-deficient cancer cells. A functional screen with an array of N-terminal and C-terminal extended SLPs pointed at the 24-mer V-SLP, elongated at the N-terminus, as most optimal vaccine candidate. This SLP was efficiently cross-presented and consistently induced a strong polyclonal LRPAP1_21–30_-specific CD8 T cells from the endogenous T cell repertoire. Thus, we designed a TEIPP SLP vaccine from the LRPAP1 signal sequence ready for validation in clinical trials.

**Supplementary Information:**

The online version contains supplementary material available at 10.1007/s00262-021-02984-7.

## Introduction

The success of T-cell-targeted immunotherapy relies on the presentation of tumor antigens by HLA molecules on the cell surface of cancer cells. However, cancer cells often downregulate components of the antigen-processing machinery to prevent the presentation of tumor-associated and tumor-specific antigens by HLA class I molecules [1–3]. One critical step in this intracellular process is the transport of liberated peptides over the ER membrane by the dedicated pump TAP, which functions as a bottleneck and delivers peptides for all HLA class I alleles [2–4]. Such antigen presentation defects allow tumors to escape from (checkpoint induced) CD8 T cell immunity and are frequently observed in human cancers [5–8]. We previously described a novel subset of tumor antigens that are selectively presented by cancers with down modulated TAP expression and called these TEIPP (tumor epitopes associated with impaired peptide processing) [9–13]. In a recent study, we reported on the molecular identification of 16 HLA-A2 presented human TEIPP antigens by means of a novel hybrid forward-reverse approach [14, 15]. One of the identified antigens derived from the signal sequence of the LRPAP1 protein and was presented on multiple TAP-deficient cancer types, including renal cell carcinoma, lymphoma, melanoma, and colon carcinoma. Moreover, LRPAP1_21–30_-specific CD8 T cells were present in the natural repertoire of all tested HLA-A2 positive healthy donors and in vitro stimulated T cells were capable of targeting these HLA class I^low^ immune-escaped cancers.

We now aim to exploit TEIPP antigens for the benefit of cancer patients and develop therapeutic formats for immunotherapy. Although interest in cancer vaccines waned long ago due to a sheer lack of objective clinical responses in hundreds of trials, they recently regained attention since novel platforms demonstrated efficacy to induce broad CD4 and CD8 anti-tumor T cell immunity, increase immune infiltration of human cancers and eradicate premalignant lesions [16–18]. Moreover, addition of therapeutic cancer vaccines to standard-of-care chemotherapy or immune checkpoint therapy improved the overall response rate and median overall survival in cancer patients [19–21].

All T-cell-geared vaccination platforms depend on delivery of tumor antigens to the host and on the exceptional capability of dendritic cells to cross-present these tumor antigens in HLA class I and II molecules for subsequent T cell activation. Many parameters are important for successful development of therapeutic cancer vaccines, including delivery systems, route of administration and adjuvants, which are supposed to activate the innate immune system and induce T cell co-stimulatory molecules. Our group developed the synthetic long peptide (SLP) vaccination platform and showed that long peptides (20–35 amino acids) possess the capacity to trigger CD4 and CD8 T cell responses and result in eradication of premalignant lesions [16, 17, 22]. Cross-presentation of long peptides by host dendritic cells depends on processing via the proteasome, the dominant proteolytic enzyme for the generation of antigenic peptides, and TAP-mediated transport resulting in HLA class I loading [23–26]. However, TEIPP tumor antigens are per definition processed in an alternative, TAP-independent way [15, 27–31]. TEIPP epitopes can access the HLA class I loading machinery via liberation by the signal peptidase (SPase) and others depend on intramembranous cleavage by signal peptide peptidase (SPPase) [27, 29]. Thus, TEIPP antigens might need modifications in order to be exploited as SLP.

Here, we describe the pre-clinical development of the most prominent human TEIPP antigen derived from the LRPAP1 protein [15]. In short, we demonstrate that a single amino acid exchange at the C-terminus of this peptide-epitope allows application in the SLP platform. The alteration resulted in a higher binding affinity to HLA-A2, a better proteasome cleavage prediction, and a more efficient cross-presentation by monocyte-derived dendritic cells. An in vitro vaccination protocol led to the generation of CD8 T cultures that exhibited an indistinguishable reactivity to the altered peptide sequence and the natural peptide as well as a highly selective capacity to recognize TAP-deficient tumor cells. These data indicate that small alterations to CD8 T cell epitopes derived from signal sequences enable their cross-presentation from the SLP format and the subsequent induction of functional CD8 T cell responses from the natural T cell repertoire.

## Materials and methods

### Cell culture

Tumor cells were cultured in DMEM medium (Gibco) supplemented with 100ug/mL streptomycin, 100 U/mL penicillin, 2 mM L-glutamine (Invitrogen) and 10% FCS (Gibco). Genetic TAP1 and LRPAP1 knockout in human cancer cell lines was generated by CRISPR/CAS9 as described before [15]. TAP1 and LRPAP1 qPCR protocol was previously described [15]. Human T cells were cultured in IMDM medium (Gibco) supplemented with 2 mM L-glutamine, 10% human serum (Sanquin Bloodbank, Amsterdam), and 50 IU/mL IL-2 (Proleukin, Novartis). LRPAP1-specific T cell clone 1A8 was cultured by stimulation mixture of 5 × 10^6^ T cells, 800 ng/ml PHA (phytohaemagglutinin) (Murex Biotech), IL-2 (100 U/mL), IL-7 (5 ng/mL), and a feeder mix containing irradiated PBMCs (5 × 10^6^ cells, 50 Gy), and EBV-JY cells (5 × 10^5^ cells, 75 Gy) in 5 mL T cell medium per T25 flask every 10 to 14 days. T cells were split with T cell medium to T75 flasks. All cells were maintained in a humidified air incubator at 37 °C and 5% CO_2_.

### Cross-presentation and direct presentation assays

Monocyte-derived dendritic cells (MoDC) were generated as previously described [32]. In short, HLA-A*02:01 positive PBMCs were isolated from buffy-coats from consented donors (Sanquin Bloodbank, Amsterdam), using a gradient ficoll layer. PBMCs were incubated with anti-CD14 magnetic beads (Miltenyi) for 20 min at 4 °C and the CD14 positive monocytes were isolated using magnetic separation columns (Miltenyi). CD14 + monocytes were cultured in RPMI medium supplemented with 10% FCS, GM-CSF (800 U/mL), and IL-4 (500 U/mL) for 6 days to generate immature MoDC. On day 6, the immature moDCs were incubated with synthetic long peptides (20 µg/mL, in house synthesized) for 24 h, and matured with LPS (20 ng/mL) on day 7. Differentiation of monocytes to matured moDCs was verified by flow cytometry analysis by upregulation of CD86 and HLA-DR. Matured moDCs were washed and co-cultured with previously established LRPAP1-specific CD8 T cell clone 1A8 for 16 h [15]. Cytokine release by T cells was then measured in supernatants by ELISA, as previously described [15]. Direct presentation of synthetic long peptides was tested by continuous incubation with LRPAP1-specific CD8 T cell clone 1A8 for 16 h in the absence of moDC. Cytokine release by T cells was then measured in supernatants by ELISA.

### Peptide-specific T cell expansion and in vitro vaccination

For isolation and expansion of LRPAP1-specific CD8 T cells, HLA-multimer enriched T cells from HLA-A*02:01 positive PBMCs of buffy-coats were co-cultured with short synthetic peptides FLGPWPAAS or FLGPWPAAV (1 µg/mL), IL-2 (100 U/mL), IL-7 (5 ng/mL), and a feeder mix containing irradiated PBMCs (2 × 10^6^ cells, 50 Gy), and EBV-JY cells (2 × 10^5^ cells, 75 Gy) per well in a 24-well plate.

In the in vitro vaccination protocol, mature moDC were pre-incubated with synthetic long peptides (20 µg/mL, in house synthesized) and then co-cultured with HLA-multimer enriched T cells from HLA-A*02:01 positive PBMCs of buffy-coats. T cell bulks were stimulated a second time on day 14. T cell specificity and reactivity were analyzed by flow cytometry.

### T cell clone isolation from expanded T cell bulks

Expanded CD8 T cells were single-cell-sorted on HLA-multimer-positive cells in 96-well plates, using an Aria III FACS machine (Becton Dickinson). Following sorting, single T cells were stimulated using PHA (800 ng/mL), a feeder mix containing irradiated PBMCs (1 × 10^5^ cells, 50 Gy), and EBV-JY cells (1 × 10^4^ cells, 75 Gy), supplemented with IL-2 (100 U/mL) and IL-7 (5 ng/mL) every 10–14 days. Expanded T cell clones were analyzed on HLA-multimer specificity and further expanded in T25 culture flasks using the PHA expansion protocol.

### Flow cytometry analysis

CD8 T cells were harvested and incubated with HLA-multimers for 15 min at 4 °C and washed three times with cold PBS/BSA prior to staining with antibodies. Cells were incubated with anti-CD3 (clone SK-7, BD), anti-CD4 (clone SK-3, BD), anti-CD8 (clone SK-1, BD) antibodies for 30 min at 4 °C and washed three times with cold PBS/BSA. T cell activation was measured by intracellular IFNγ staining (XMF1.2, BioLegend) using an intracellular cytokine staining kit (BioLegend) according to manufactures protocol. moDC’s were stained with anti-CD1a (clone HI149, BD), anti-CD14 (clone M5E2, BD), anti-CD80 (clone L307.4, BD), anti-CD83 (clone HB15e, BD), anti-CD86 (clone IT2.2, BioLegend), and anti-HLA-DR (clone G46-6, BD) antibodies for 30 min at 4 °C and washed three times with cold PBS/BSA. Samples were acquired using a BD LSRFortessa™ flow cytometry machine and analyzed using FlowJo software (Tree Star).

### Statistics

Statistical analysis was performed in GraphPad Prism software (version 9) using student t tests (paired, two-tailed) with welch correction to determine the statistical significance of the differences. All experiments were performed at least two times. Differences were considered statistically significant at *p* < 0.05. (*p < 0.05, **p < 0.01, ***p < 0.001).

## Results

### LRPAP1_21–30_-specific T cells are present in lung cancer patients and recognize TAP-deficient human cancer cells

**Fig. 1:**
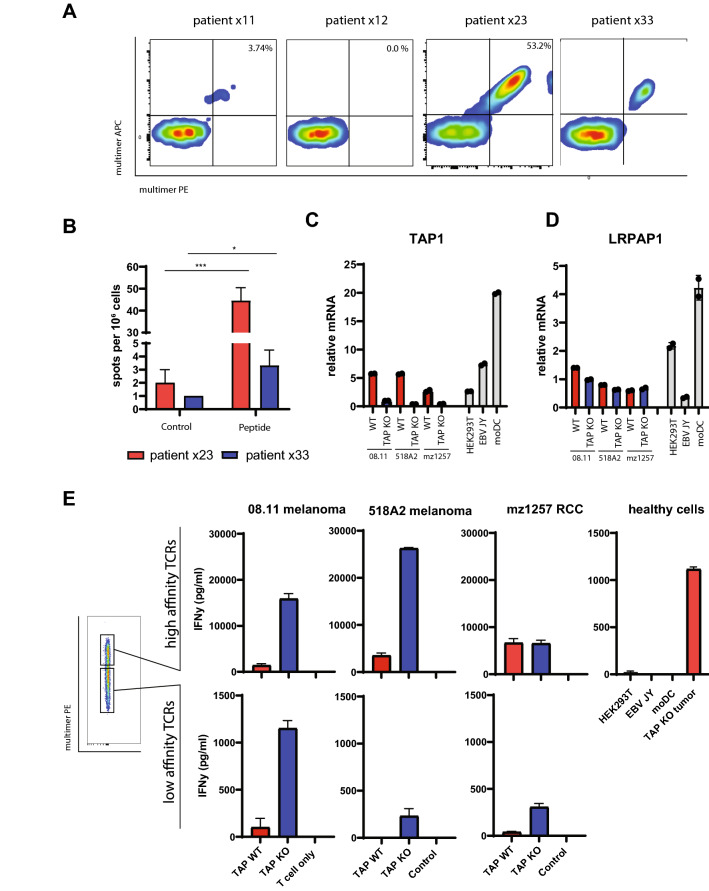
Functional LRPAP1-specific T cell exists in the repertoire of non-small cell lung adenocarcinoma patients. **a** PBMCs from 4 different patients were enriched for LRPAP1_21–30_-specific CD8 T cells by peptide/HLA-A2 multimer magnetic bead isolation and stimulated with short peptide. Flow cytometry analysis was used to visualize the peptide-specific T cell frequencies with two different fluorescent labels on the multimers. **b** ELISPOT assay for IFNγ release by T cell bulks upon recognition of the LRPAP1 peptide. Pictures of the ELISPOT plate are depicted in Supplementary Figure 1. **c**, **d** Relative gene expression of TAP1 (**c**) and LRPAP1 (**d**) of a panel of 3 human cancer lines (wild type versus TAP-KO), and 3 non-transformed cells. **e** LRPAP1_21–30_-specific polyclonal T cell culture of patient × 23 was sorted in high- and low-affinity T cells on basis of peptide/HLA-A2 multimer staining intensity and tested against the human cell panel. Cytokine concentrations were measured by IFNy ELISA

Our study on LRPAP1_21–30_-specific CD8 T cells was thus far limited to T cell repertoires of healthy donors. We thus first set out to assess the existence of LRPAP1_21–30_-specific T cells in patients with cancer. PBMCs from four HLA-A*0201 positive non-small cell lung adenocarcinoma were selected from a previous clinical study [33]. Three of these patients had stage I/II disease (× 12, × 23, and × 30) and were chemo- and radiotherapy naïve, while one patient (× 11) had stage IV disease and was treated with chemotherapy more than 3 months before PBMC isolation. None of the patients received checkpoint inhibitors. PBMCs were enriched for LRPAP1_21–30_-specific T cells by HLA-multimer magnetic bead isolation, followed by three rounds of peptide stimulation, according to our previously developed protocol [15]. LRPAP1_21–30_-specific CD8 T cells were detected in three out of four patients, as shown by clear populations of HLA-multimer staining T cells (Fig. 1a, Table 1). Half of the T cell culture from patient × 23 consisted of LRPAP1_21–30_-specific T cells after three in vitro stimulations, pointing to a very efficient enrichment of this specificity. Expanded T cell bulks of patient × 23 and × 33 were tested for reactivity against the peptide in ELISPOT assay and IFNγ release from these bulk cultures validated the functionality of these T cells (Fig. 1b, Supplementary Figure 1). Next, we examined the capacity of × 23 patient-derived T cells to recognize TAP-deficient human cancers cells. A panel of three human HLA-A*0201 cancer cell lines (08.11 and 518A2 melanomas and mz1257 renal cell carcinoma) and three HLA-A*0201 non-cancer cell lines (HEK293T, EBV-JY, and moDC) were selected. Cancer cell lines expressed relatively low levels of TAP1 transcripts, but were rendered completely negative by CRISPR-CAS9 technology (Fig. 1c). LRPAP1 expression was similar between cancer cell lines and healthy cells, with moDC having approximately four times higher levels than the cancer lines (Fig. 1d). We then sorted the × 23 T cell bulk into two fractions on basis of the HLA-multimer staining intensity and tested their reactivity against this cell panel (Fig. 1e). The high-intensity stained LRPAP1_21–30_-specific T cells responded strongly to TAP-deficient cancer cells, but not to the LRPAP1-positive healthy cells. The low intensity stained LRPAP1_21–30_-specific T cells also selectively recognized TAP-deficient cancer cells, but to much lower extent, considering the difference in IFNγ release. This reactivity pattern of selective targeting TAP-deficient cancer cells is characteristic for TEIPP specificity, as the target protein is expressed by healthy cells but the derived peptide-epitope is not presented by HLA class I at their cell surfaces due to competing peptide repertoires [34, 35]. We concluded that patients affected with lung cancer harbor exploitable TEIPP-specific T cells in their CD8 T cell repertoire.

**Table 1:** LRPAP1-specific CD8 T cells in lung cancer patients

Patient	Multimer staining	T cell recognition	
		Peptide	Tumor
× 11	+	nd^1^	nd
× 12	−	nd	nd
× 23	+ +	+ +	+ +
× 33	+	+	+

^1^Not determined

### The LRPAP1 signal peptide is cross-presented as a synthetic long peptide after amino acid substitution of the C-terminus

**Fig. 2:**
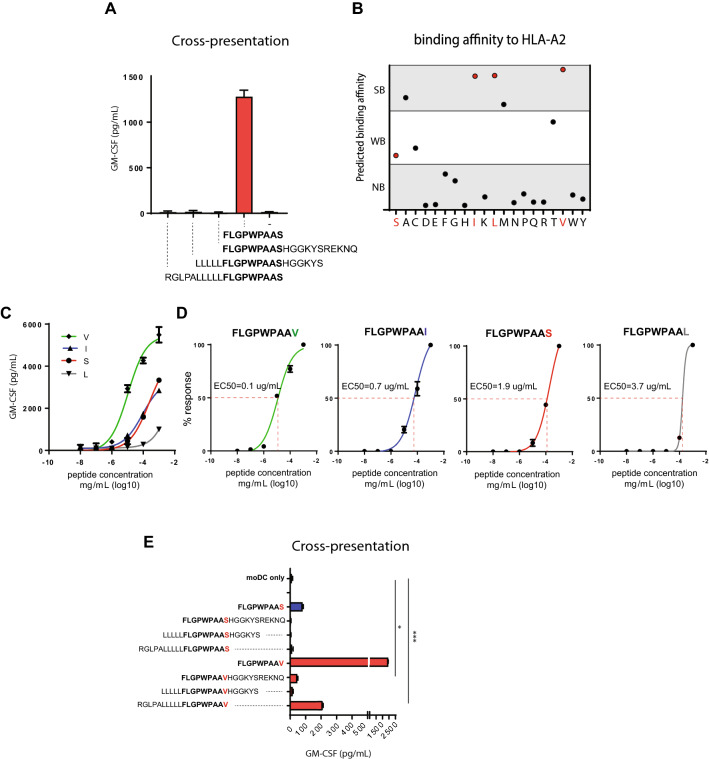
Amino acid replacements at the C-terminus of the LRPAP1 signal peptide. **a** Long peptides of the LRPAP1 epitope FLGPWPAAS were incubated with monocyte-derived dendritic cells and examined for cross-presentation with TEIPP-specific CD8 T cell clone 1A8. Natural flanking amino acids were used to elongate the minimal epitope. Exogenous pulsing with short peptide was used as positive control. **b** Predicted HLA-A*0201 binding affinity scores of LRPAP1 peptides with substituted amino acids at the C-terminal p9 (netMHC 4.1 algorithm). Each dot represents one peptide. Strong binder (SB, < 50 nM), weak binder (WB, 50 < 500 nM), non-binder (NB, > 500 nM), see Supplementary Table 1 for exact values and ranking. Red dots represent the peptide variants included in the follow-up experiments. **c** Functional T cell avidity was estimated by activation of LRPAP1_21–30_-specific T cell clone. The LRPAP1 short peptide variants were pulsed on HLA-A*0201 EBV-JY B cells in serial dilutions of the peptides. **d** EC50 values were calculated from values obtained in C. **e** Long peptides of the S- and V- variants of the LRPAP1 sequence were incubated with monocyte-derived dendritic cells and examined for cross-presentation with TEIPP-specific T cell clone 1A8. Pulsed short peptide served as positive controls. Means with SD are plotted from a representative experiment (*n* = 3)

We then set out to design a synthetic vaccine capable to raise LRPAP1_21–30_-specific CD8 T cell responses in patients. Over the last decennia, we developed the synthetic long peptide (SLP) platform and successfully applied therapeutic SLP vaccination for HPV-induced cancers [17, 19, 21]. Therefore, we first assessed the efficiency of cross-presentation of long versions of the signal peptide LRPAP_21–30_ in dendritic cells, using natural flanking amino acids extending the amino-terminus, the carboxy-terminus, or both ends. Monocyte-derived dendritic cells (moDC) were incubated with these three SLPs or with the minimal peptide-epitope, then matured, and used as targets for the previously [15] isolated CD8 T cell clone 1A8 to assess correct processing and HLA-A2 presentation of the minimal TEIPP epitope. None of the SLP variants were cross-presented to T cells, whereas exogenous pulsing of the short LRPAP_21–30_ peptide did stimulate the T cells (Fig. 2a). These results suggested that cross-presentation of the LRPAP_21–30_ epitope from its longer peptide stretch is not efficient and had to be optimized for vaccine applications.

Cross-presentation of long peptides by dendritic cells involves multiple sequential steps, including uptake via endocytosis, cytosolic cleavage of the SLP into short peptides by the proteasome, transport over the ER membrane by TAP and loading onto MHC-I molecules [22, 23]. Previous studies have shown that the LRPAP_21–30_ epitope has a moderate binding affinity for HLA-A*0201, since the C-terminal serine is not a preferred anchor residue for this allele [15]. The C-terminus docks into the F-pocket and is important for binding to MHC-I molecules, but does not directly interact with T cell receptors (TCR) and might thus be replaced. We investigated if exchange into another amino acids would result in a more efficiently processed epitope. First, the binding affinities to HLA-A*0201 of all peptide sequences with alternative amino acids at position 9 were estimated using an in silico algorithm (Fig. 2b and Supplementary Table 1). The C-terminal serine (S) had a weak predicted binding- and ranking-score (WB) (affinity = 364 nM, % rank = 2.50, respectively). However, substitution to isoleucine (I), leucine (L), or valine (V) resulted in strongly improved predicted binding affinities of 12 nM, 11 nM, and 6 nM, respectively, categorizing these peptides as strong binders (SB). Second, we examined whether these substitutions would interfere with T cell receptor interaction. Short minimal peptides with the C-terminal isoleucine, leucine, or valine were exogenously pulsed on HLA-A*0201 positive T2 cells and T cell activation of clone 1A8, which was isolated against the natural S-variant, was measured. The I- and L-variant peptides induced similar or worse cytokine responses when compared to the natural S-containing peptide, suggesting that T cell interaction was disturbed despite stronger binding to HLA-A2 molecules. In contrast, the V-peptide induced a more potent IFNy response (Fig. 2c). Calculation of the EC50 values confirmed that the V-peptide variant elicited the strongest T cells response at limiting peptide concentrations (EC50 in ug/mL = S: 1.9, I: 0.7, L: 3.7, V: 0.1) (Fig. 2d). We concluded that substitution of serine (S) to valine (V) at the C-terminus of the LRPAP_21–30_ peptide allowed for proper TCR interaction and resulted in a 19-fold better T cell activation. Finally, cross-presentation of the V-variant was evaluated as SLP extended with natural flanking amino acids. After uptake and processing, moDCs were co-cultured with the LRPAP1_21–30_-specific T cell clone 1A8 and cytokine production was measured (Fig. 2e). Whereas the natural S-SLP variants failed to activate the T cell clone, the N-terminal extended V-SLP peptide, and to a lesser degree also the C-terminal variant, were processed and presented by the moDCs (Fig. 2e).

**Fig. 3:**
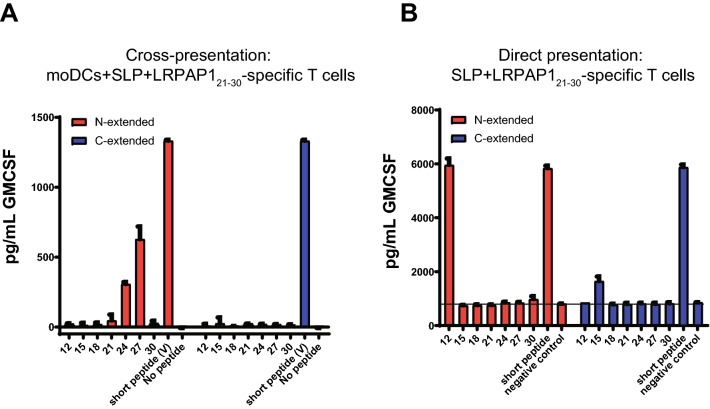
C-terminal amino acid exchange of the LRPAP1 epitope allows cross-presentation of the long peptides by dendritic cells. **a** N-extended and C-extended length variants of the V-SLP were tested for cross-presentation with monocyte-derived dendritic cells and T cell clone 1A8. **b** N-extended and C-extended length variants of the V-SLP were directly loaded on T cell clone 1A8 for recognition without processing via monocyte-derived dendritic cells. Cytokine release was measured by ELISA. Means and SD are plotted from a representative experiment (*n* = 3) in all panels

We then evaluated the most optimal length of the V-SLP in this system. Seven different length variants that were extended at their N- or C-terminus of the V-SLPs (Table 2) were examined in the aforementioned cross-presentation assay (Fig. 3a). Interestingly, the 24-mer and 27-mer V-SLPs, extended at the N-terminus were processed by moDC. The somewhat longer 30-mer surprisingly failed to be cross-presented. None of the C-terminal extended variants were efficiently cross-presented, indicating the absence of necessary proteolytic enzymes to liberate the C-terminus of these SLP in the cross-presentation pathway of moDC. The N-terminal extended 12-mer was the only peptide able to directly stimulate LRPAP1_21–30_-specific T cells in the absence of moDC (Fig. 3b), indicating that this length variant could induce a tolerizing T cell response in vivo rather than raising a protective T cell response [22, 25, 26]. Our data showed that the 24-mer to 27-mer, N-terminal extended, V-substituted LRPAP1 peptides are suitable for the SLP vaccination platform.

**Table 2:**
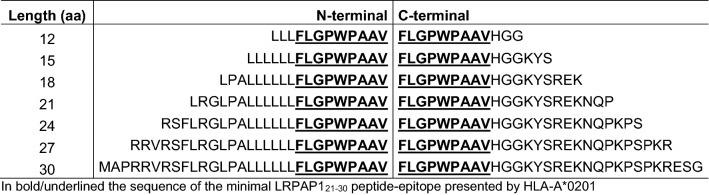
N- and C-terminal extended peptide sequences of LRPAP1 epitope

In bold/underlined the sequence of the minimal LRPAP1_21–30_ peptide-epitope presented by HLA-A*0201

### CD8 T cell repertoire isolated with the short V-peptide efficiently cross-reacts to the naturally presented S-containing LRPAP1_21–30_ epitope

**Fig. 4:**
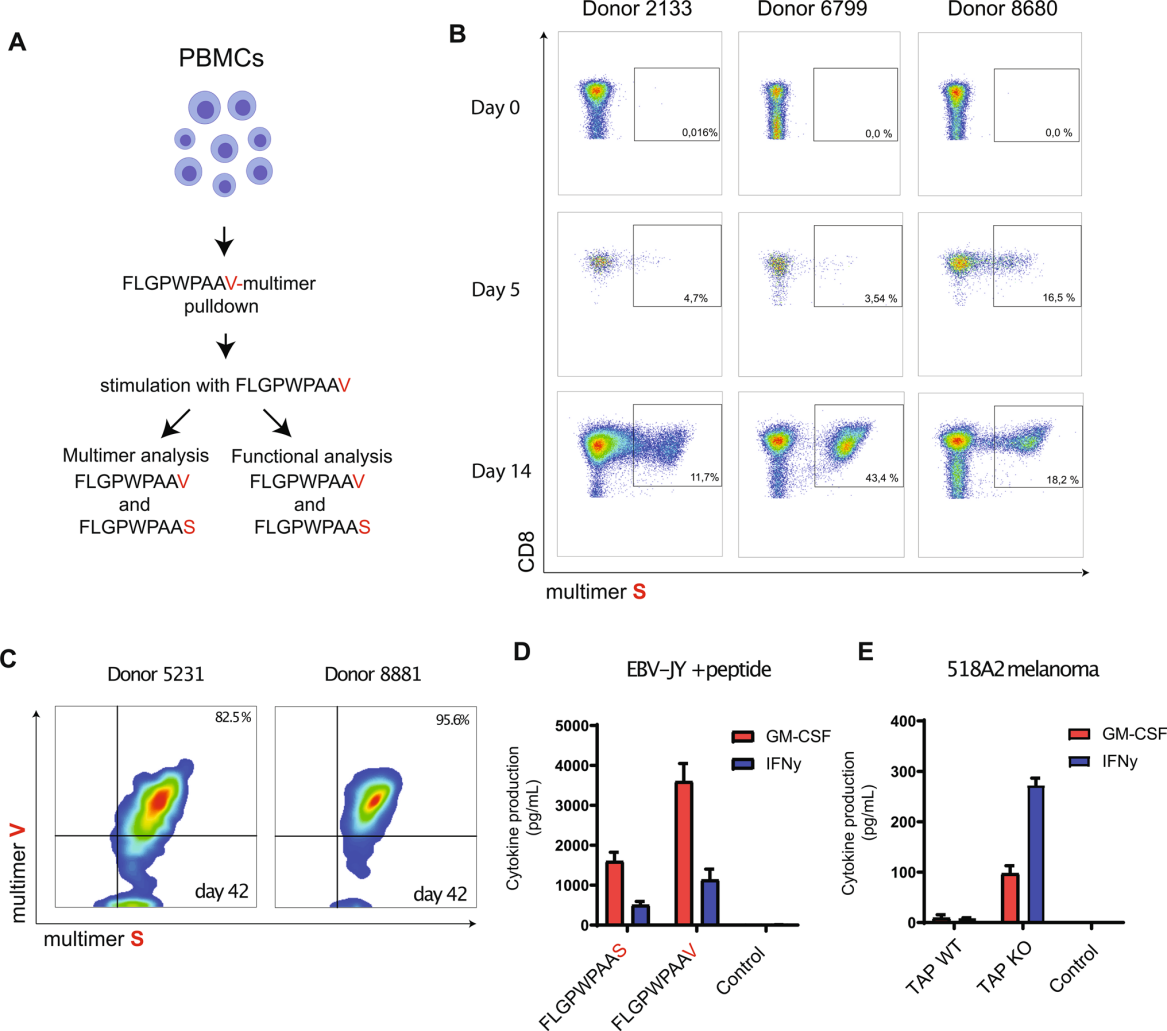
T cell repertoire isolated with the V-substituted peptide targets the natural peptide-epitope. **a** Schematic overview of the tetramer pull-down approach and subsequent stimulation with short peptides. **b** Frequencies of S-variant/HLA-A2 multimer stained CD8 T cells in short-term polyclonal T cell bulk cultures induced with FLGPWPAA**V**-peptide. **c** Binding of both S-variant/HLA-A2 and V-variant/HLA-A2 multimers on long-term CD8 T cell cultures induced with FLGPWPAA**V**-peptide. **d** Reactivity of polyclonal T cell bulk isolated using the V-peptide variant against V-peptide and natural S-peptide pulsed on EBV-JY B cells. **e** Reactivity of one T cell culture to WT and TAP knockout 518A2 melanoma cells. Means and SD are plotted of one out of three independent experiments in all panels. Three different healthy donors were examined yielding similar results

Next, we evaluated the CD8 T cell repertoire that can be raised using the V-peptide variant and if these T cells cross-react to the wild-type LRPAP1_21–30_ peptide, which is naturally presented by TAP-deficient immune-escaped cancer cells. Therefore, CD8 T cell cultures were generated using our previously described approach with HLA-A*0201 tetramer pull-down and subsequent expansion by stimulations with short V-containing peptide [15] (Fig. 4a). This short-term culture protocol with the V-variant resulted in the generation of polyclonal CD8 T cell bulks containing cells displaying specificity for the natural LRPAP1_21–30_ peptide (Fig. 4b, c). These V-variant stimulated CD8 T cells were present in short-term bulk cultures from five out of five healthy donors. The frequency of LRPAP1-specific CD8 T cells increased over time, indicating that the V-peptide stimulated the selective expansion of peptide-specific T cells from the endogenous repertoire (Fig. 4b). Repeated stimulations with the V-containing peptide resulted in high frequencies displaying binding capacity of the V-peptide multimer as well as the natural S-peptide multimer (Fig. 4c). Both HLA-A2 multimers bound with similar intensity on a per cell basis, indicating that T cells raised against the V-variant were fully capable to interact with the S-peptide (Fig. 4c). Vice versa, CD8 T cell bulk cultures raised against the natural short S-variant also bound both multimers with similar intensity (Supplementary Figure 2A). These data imply that the exchanged C-terminus into a valine did not alter the conformation of this peptide/HLA complex with respect to the TCR interaction sites.

To test the functionality of the V-variant stimulated T cell bulks, we measured cytokine responses toward short peptides pulsed on immortalized HLA-A*0201-positive B-LCL cells and TAP-deficient melanoma cells (Fig. 4d, e). V-variant stimulated T cell bulks responded to both the wild-type and variant peptides by secretion of IFNy and GM-CSF and, moreover selectively targeted TAP-deficient melanoma cells, typical for TEIPP specificity. These data indicated that the T cell repertoire raised by the high-affinity binding V-peptide is functionally cross-reactive to the naturally presented S-peptide. Very similar results were obtained for S-induced T cell bulk cultures (Supplementary Figure 2B, C). Thus, this amino acid exchange results in a peptide variant capable of selecting LRPAP1_21–30_-specific T cells from the total CD8 T cell repertoire of healthy donors.

### In vitro vaccination with V-SLP promotes the expansion of LRPAP1_21–30_-specific TEIPP T cells

**Fig. 5:**
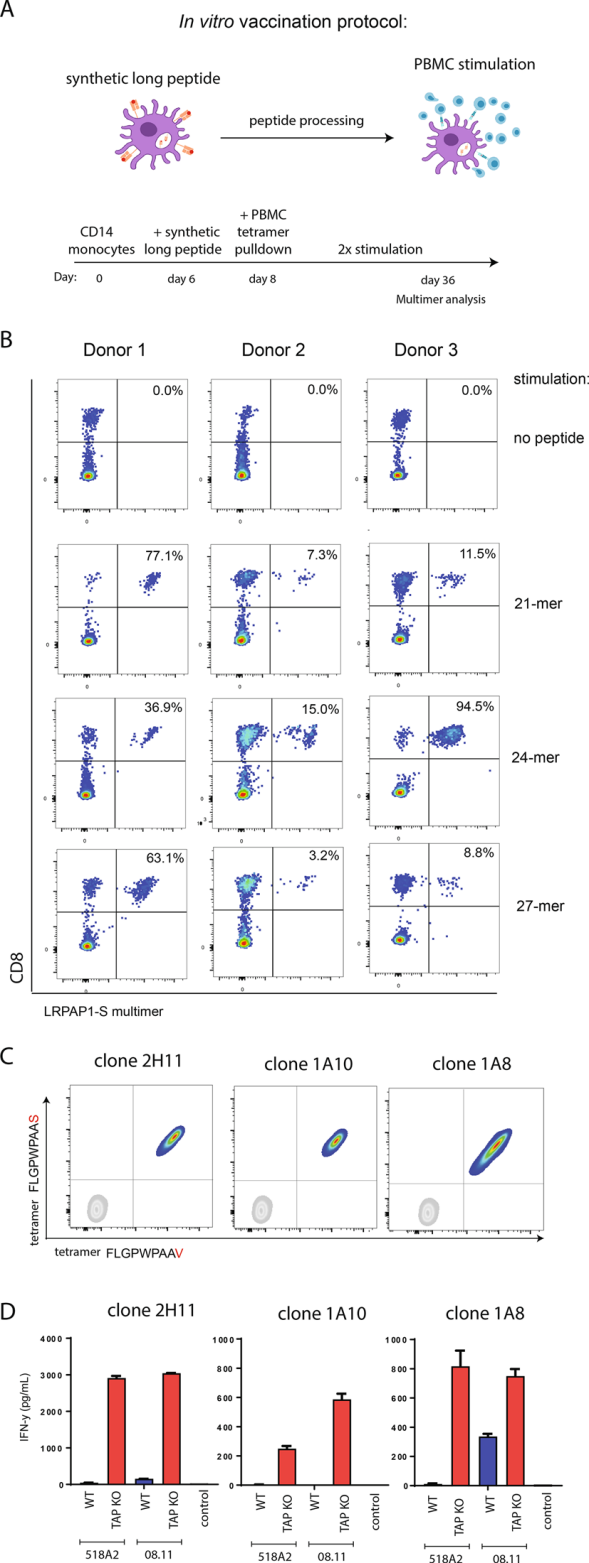
In vitro vaccination with LRPAP1 V-SLP results in expansion of tumor-reactive TEIPP-specific T cells. **a** Schematic overview of the in vitro vaccination protocol using V-variant synthetic long peptides for priming of CD8 T cells from PBMC of healthy donors. **b** Different N-terminal extended length variants (18-mer to 27-mer) of the V-SLP were co-cultured with moDCs from three different donors. The capacity to cross-present the epitope and to induce expansion of peptide-specific T cells was analyzed by measuring the frequencies of LRPAP1_21–30_-specific CD8 T cells by peptide/HLA-A2 multimer staining. As control, moDCs were incubated with PBMC without long peptide. Representative data out of three experiments is shown. **c** Two LRPAP1-specific T cell clones (2H11 and 1A10) were generated from SLP-induced T cell bulks by single-cell sorting. LRPAP1 specificity was measured by flow cytometry. Clone 1A8 was isolated from a previous study [15], and used as a reference clone. **b** LRPAP1-specific T cell clones were tested for reactivity against TAP-KO cancer cells and their WT counterparts. Clone 1A8 was used as a reference T cell clone. Means and SD are shown from one out of two experiments with similar outcome

So far, cross-presentation of long peptide variants was examined with the use of TEIPP T cell clone 1A8. The in vitro vaccination protocol was used to validate the concept of V-exchanged SLP vaccination for de novo induction of TEIPP T cell responses [32, 36]. This protocol mimics the in vivo situation of therapeutic vaccines as moDCs are incubated with SLP, an adjuvant and autologous T cells (Fig. 5a). After two rounds of SLP stimulation, a great expansion of LRPAP1_21–30_-specific CD8 T cells was observed in three different PBMC donors, whereas no specific T cells were induced with non-loaded control moDC (Fig. 5b). The 24-mer V-SLP peptide RSFLRGLPALLLLLLFLGPWPAAV raised most consistently strong T cell responses in three independent donors, whereas the 27-mer was less effective in this assay. Interestingly, our previous results demonstrated that all LRPAP1_21–30_-specific CD8 T cells were still in the naïve state in healthy donors [15], indicating an efficient de novo priming in this in vitro vaccination protocol.

Next, we generated CD8 T cell clones from these polyclonal T cells bulks and determined their TEIPP reactivity. Multimer-positive T cells were sorted by flow cytometry as single cells and expanded in an antigen-unrelated manner using phytohaemagglutinin (PHA). More than ten CD8 T cell clones were isolated and all showed high and equal staining intensity for both V-peptide and S-peptide multimers, as displayed for the two examples 2H11 and 1A10 (Fig. 5c). Finally, these two clones efficiently and selectively recognized two TAP-deficient melanomas in a manner very similar to the previously established clone 1A8 (Fig. 5d). Collectively, these results demonstrated that the V-SLP constitutes a functional TEIPP vaccine ready to be exploited for the induction of LRPAP1-specific T cell immunity.

## Discussion

The aim of this study was to design a synthetic long peptide vaccine for the TEIPP antigen derived from the LRPAP1 protein. The HLA-A*0201 presented peptide-epitope LRPAP1_21–30_ (FLGPWPAAS) is encoded by the signal peptide, a domain that docks protein translational products to the sec61 translocation channel in the ER membrane [37]. These signal peptides are usually cleaved from nascent proteins by the protease Signal Peptidase (SPase), resulting in small protein transmembrane remnants, liberated from the ER membrane by Signal Peptide Peptidase (SPPase) which cleaves within the lipid bilayer [28]. This proteolysis results in the TAP-independent routing of the C-terminal domain of signal peptides and explains the overrepresentation of signal peptides, like LRPAP1_21–30_, in the HLA class I peptidome of TAP-deficient cells [38–40]. Previous studies demonstrated that signal peptides are poorly cross-presented by host dendritic cells of tumor-bearing mice [41], suggesting that this antigenic material from dying tumor cells is not suitable for processing after uptake by dendritic cells. Most studied cross-presentation systems demonstrate a dependency on proteasomes, which dominantly contribute to liberation of the HLA class I immunopeptidome [4]. Synthetic long peptide vaccines are generally also processed via the classical cytosolic route, involving proteasome-TAP pathway [42], as these long peptides end up in the cytosol of dendritic cells after co-incubation [23, 24]. Here, we report that a signal sequence-derived peptide from the LRPAP1 protein is efficiently cross-presented as long peptide in dendritic cells after a single amino acid exchange of its C-terminal anchor residue. The fact that one single amino acid substitution from serine (S) to a valine (V) at p9 of the LRPAP1 peptide rendered the long peptide sensitive for cross-presentation by moDC (Figs. 2, 3) might be related to higher HLA-A2 binding stability or, alternatively, to difference in uptake or routing of the peptide.

Head-to-head comparison of T cell repertoires isolated with short S- or V-peptides revealed that both repertoires were comparable concerning reactivity and functionality (Fig. 4). Moreover, stimulation with dendritic cells loaded with long peptides, which require intracellular cross-presentation, resulted in polyclonal CD8 T cell bulks and clones with high affinity and strong capacity to recognize the natural S-variant on TAP-deficient melanomas (Fig. 5). These findings imply that the V exchange does not alter the conformation of the peptide/HLA-A2 complex and, importantly, that vaccination with the optimized V-SLP will result in the generation of LRPAP1_21–30_-specific effector T cells with high-affinity TCRs. The advantage of SLP over vaccination with short minimal epitopes is that SLP is selectively presented by professional antigen-presenting cells, e.g., dendritic cells, due to their cross-presentation requirement. Dendritic cells are critical for proper priming of T cell responses. In contrast, short peptides are also presented by non-professional antigen-presenting cells, e.g., B cells, via exogenously loading, which results in tolerizing T cell responses [22, 25, 26]. Interestingly, the netCHOP algorithm for proteasome cleavage prediction reveals a very high score (> 0.9) for cleavage within the leucine stretch at the N-terminal region of our long LRPAP1 peptide. This suggests that our optimal TEIPP SLP of 24 amino acids is processed and cross-presented via proteasomes in dendritic cells and therefore capable of inducing CD8 T cell immunity.

Interestingly, we previously showed that LRPAP1_21–30_-specific T cells in healthy blood donors all reside in the naïve repertoire, implying that our in vitro vaccination protocol actually primed CD8 T cells and not merely reactivated memory T cells [15]. The differentiation state of T cells in cancer patients and in particular those harboring TAP-deficient tumor cells needs further analysis, however, data from our mouse tumor model revealed that TEIPP-directed CD8 T cells are even naïve under these circumstances [9, 43]. We found that TAP-deficient tumors failed to prime TEIPP T cells and also host dendritic cells were unable to pick up TEIPP antigens and cross-prime them. Consequently, TEIPP immunity might need to be installed by active immunizations, like we suggested here via SLP vaccines. Thus, the optimized long peptide of the signal peptide of LRPAP1 containing one amino acid exchange constitute an ideal vaccine candidate to induce TEIPP immunity in HLA-A2 cancer patients. Although cancer vaccines lack potential to induce cancer remission as standalone therapy [16], combinations with checkpoint blockade or other immunomodulatory compounds might lead to successful immune attack to immune-escaped TAP-deficient cancers, as has been shown for the combination of cancer-virus vaccination with PD-1 blockade [19].

### Supplementary Information

Below is the link to the electronic supplementary material.

Supplementary file1 (DOCX 13 kb)

Supplementary file2 (PDF 1076 kb)

Supplementary file3 (PDF 1231 kb)
