# Supplementary material for: Cross-presentation of a TAP-independent signal peptide induces CD8 T immunity to escaped cancers but necessitates anchor replacement
Source: Cancer Immunol Immunother. 2021 Jun 17;71(2):289–300. doi: 10.1007/s00262-021-02984-7 (PMC8783882; doi:10.1007/s00262-021-02984-7)
Supplement: Supplementary file 1 — Supplementary file1 (DOCX 13 kb) [file 262_2021_2984_MOESM1_ESM.docx]

SUPPLEMENTARY FIGURE LEGENDS:

Supplementary figure 1: Images of the ELISPOT plate with polyclonal T cell cultures from patient X23 and X33. T cell bulks were stimulated with short LRPAP1 peptide or medium as control.

Supplementary figure 2: T cell cultures isolated and stimulated with natural short S-variant peptide. (A) HLA-A2 multimer staining of the polyclonal T cell repertoire isolated by S-peptide multimers and subsequently stimulated with short S-containing peptide. (B) Reactivity of polyclonal T cell cultures isolated with S-peptide as measured by cytokines release upon V-peptide or S-peptide pulsed EBV-JY B-cells. (C) Reactivity of this polyclonal T cell bulk to WT and TAP-KO 518A2 melanoma cells. Means and SD are plotted of one out of three independent experiments. PBMC of four donors were examined yielding similar results.

**Supplementary table 1.** HLA-A*02:01 peptide binding scores of the epitope variants

| **Peptide sequence** | **NetMHC** | | **Category** |
| --- | --- | --- | --- |
|  | **Affinity (nM)** | **Rank(%)** |  |
| FLGPWPAAS | 364.80 | 2.50 | WB^1^ |
| FLGPWPAAA | 20.23 | 0.30 | SB |
| FLGPWPAAC | 303.14 | 2.50 | WB |
| FLGPWPAAD | 6719.97 | 12.00 | NB |
| FLGPWPAAE | 4516.70 | 9.00 | NB |
| FLGPWPAAF | 521.16 | 3.00 | NB |
| FLGPWPAAG | 984.43 | 4.00 | NB |
| FLGPWPAAH | 7052.81 | 12.00 | NB |
| FLGPWPAAI | 11.98 | 0.15 | SB |
| FLGPWPAAK | 4183.99 | 8.50 | NB |
| FLGPWPAAL | 11.14 | 0.15 | SB |
| FLGPWPAAM | 27.46 | 0.40 | SB |
| FLGPWPAAN | 4995.00 | 9.50 | NB |
| FLGPWPAAP | 1762.34 | 5.50 | NB |
| FLGPWPAAQ | 3029.38 | 7.50 | NB |
| FLGPWPAAR | 3690.59 | 8.00 | NB |
| FLGPWPAAT | 66.98 | 0.80 | WB |
| FLGPWPAAV | 5.98 | 0.05 | SB |
| FLGPWPAAW | 2198.56 | 6.00 | NB |
| FLGPWPAAY | 3105.78 | 7.50 | NB |

^1^ SB, strong binder; WB, weak binder; ND, non-binder
