# Supplementary figures and images for: Cross-presentation of a TAP-independent signal peptide induces CD8 T immunity to escaped cancers but necessitates anchor replacement
Source: Cancer Immunol Immunother. 2021 Jun 17;71(2):289–300. doi: 10.1007/s00262-021-02984-7 (PMC8783882; doi:10.1007/s00262-021-02984-7)

Supplementary Figure 1.

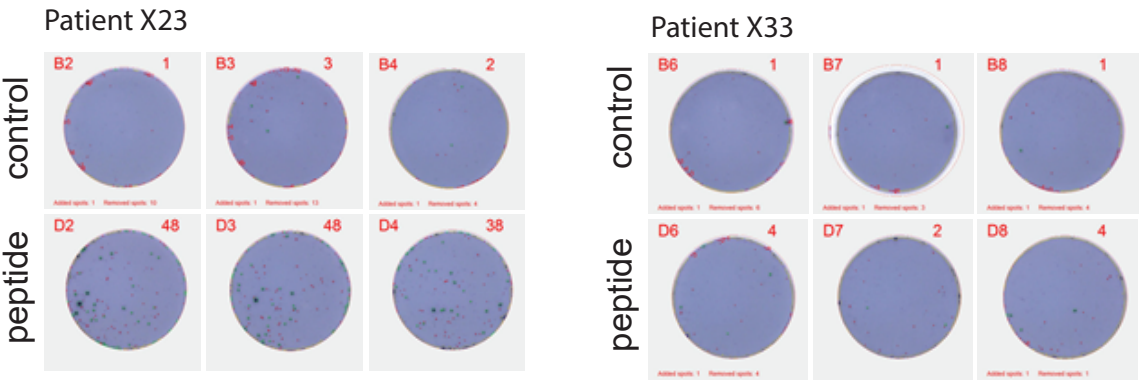

Supplement: Supplementary file 2 — Supplementary file2 (PDF 1076 kb) [file 262_2021_2984_MOESM2_ESM.pdf]

Supplementary figure 2

A

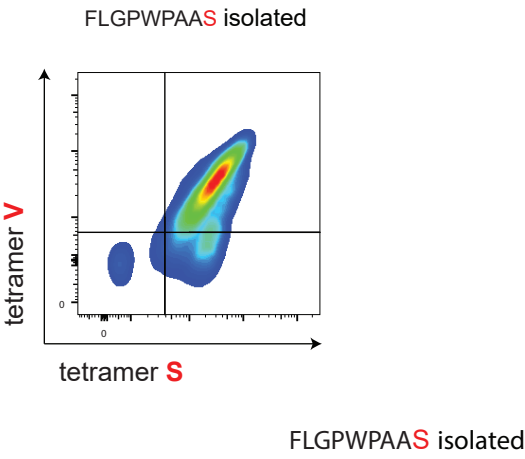

B

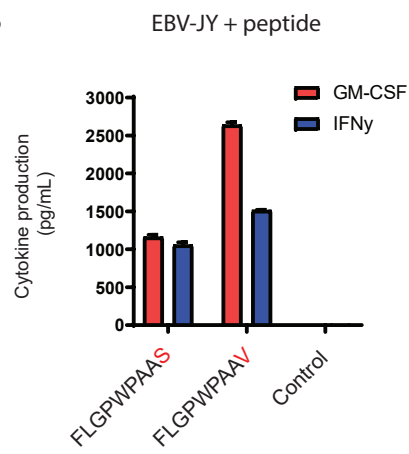

C

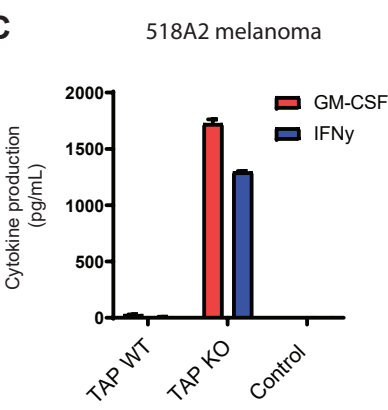

Supplement: Supplementary file 3 — Supplementary file3 (PDF 1231 kb) [file 262_2021_2984_MOESM3_ESM.pdf]
